# A diagnostic bias might be a much simpler explanation for the apparently elevated risk for nasopharyngeal cancer with respect to formaldehyde

**DOI:** 10.1186/s12995-016-0143-4

**Published:** 2016-12-05

**Authors:** Matthias Möhner, Andrea Wendt

**Affiliations:** Federal Institute for Occupational Safety and Health, Division Work and Health, Nöldnerstr. 40-42, 10317 Berlin, Germany

**Keywords:** Formaldehyde, Nasopharyngeal cancer, Cohort study, Standardized mortality ratio, Diagnostic bias

## Abstract

In 2009, a working group of the International Agency for Research on Cancer classified formaldehyde as carcinogenic to humans (Group 1) and concluded that formaldehyde causes cancer of the nasopharynx (NPC) and leukemia. The results of a large cohort study of industrial workers exposed to formaldehyde, conducted by the U.S. National Cancer Institute, mainly contributed to the available body of epidemiologic evidence. In their recent updated re-analysis of these cohort data published in your journal, Dr Marsh and his colleagues concluded that the results of the original analysis of NPC-risk are misleading because they are based on inappropriate regression analyses. In our view the reason for the elevated NPC risk reported in the original analysis might be also another one - a diagnostic bias. Therefore, it would be very helpful if the authors provided results for all other sub-categories (as three-digit categories of the International Classification of Diseases) of the pharynx to verify the hypothesis described and, hence, to clarify the relationship between exposure to formaldehyde and the risk of NPC.

## Dear Editor,

In 2009, a working group of the International Agency for Research on Cancer classified formaldehyde (FA) as carcinogenic to humans (Group 1) and concluded that FA causes cancer of the nasopharynx (NPC) and leukemia [1]. The results of a large cohort study of industrial workers exposed to FA, conducted by the U.S. National Cancer Institute (NCI) [2, 3], mainly contributed to the available body of epidemiologic evidence. In their recent updated re-analysis of these cohort data published in your journal, Dr Marsh and his colleagues concluded that the results of the original analysis of NPC-risk are misleading because they are based on inappropriate regression analyses and that their updated re-analysis did not support NCI’s suggestion of a persistent association between FA exposure and NPC risk [4].

In our view the reason for the elevated NPC risk reported in the original analysis might be also another one. With regard to the International Classification of Diseases (ICD), the pharynx is the only entity for which the share of cases classified as “not specified” (PCns) is of relevant magnitude. Taking the data from the Connecticut Cancer Registry – the state where the study plants are located – for the period from 2003 until 2007 and comparing the data on incidence with the corresponding mortality data results in a ratio of mortality to incidence (M/I) of 2.62 for PCns (Table 1). Moreover, the number of deaths for which NPC was stated as the underlying cause was much lower than the number of deaths for which PCns was the underlying cause. To verify this relationship we asked the Common Cancer Registry of the six eastern states of Germany to provide us with corresponding data for a certain period. Analyses for the years 2005 and 2006 indicated that M/I was in the same direction but not as pronounced as for Connecticut: 0.4 and 1.25 for NPC and PCns, respectively [personal communication with Roland Stabenow and Brigitte Streller from the Common Cancer Registry (Gemeinsames Krebsregister), Berlin, January 2016]. The reason for such a large difference might be the difficulty to get the specific diagnosis especially if the individual died at home. Hence, using nasopharynx as a single entity in a mortality study might introduce a strong diagnostic bias. A better approach would be to use the whole pharynx (ICD-8: 146–149; ICD-10: C09-C14), at least for mortality-based studies.

**Table 1:** Incidence and Mortality 2003–2007, Connecticut, Males

Entity	ICD-10	Incidence^a^		UCD^b^		MCD^c^		M/I^d^
		N	AS(W)^e^	N	ASR^f^	N	ASR^f^	
Tonsil	C09	271	2.3	26	0.29	28	0.31	0.10
Other oropharynx	C10	55	0.5	26	0.30	33	0.39	0.47
Nasopharynx	C11	63	0.6	19	0.22	24	0.29	0.30
Hypopharynx	C12-C13	128	1.1	18	0.21	21	0.25	0.14
Pharynx, unspecified	C14	21	0.2	55	0.64	71	0.83	2.62
Nose, sinuses, etc.	C30-C31	65	0.5	8	0.11	9	0.11	0.12
Pharynx	C09-C14	538	4.7	144	1.66	177	2.07	0.27

^a^- from IARC (2014) Cancer in Five Continents, Vol. X

^b^- Underlying cause of death; from CDC, National Center for Health Statistics, Underlying Cause of Death 1999–2014 on CDC WONDER Online Database, released 2015. Accessed at https://wonder.cdc.gov/ucd-icd10.html on Jan 14, 2016

^c^- Multiple cause of death; from CDC, National Center for Health Statistics, Multiple Cause of Death 1999–2014 on CDC WONDER Online Database, released 2015. Accessed at https://wonder.cdc.gov/mcd-icd10.html on Jan 14, 2016

^d^- Ratio between absolute numbers of cases for underlying cause of death and incidence

^e^- age-standardized rate (world population)

^f^- age-standardized rate (2000 U.S. Std. Population)

The results of the primary analysis of the first follow-up [5] support our hypothesis. Only one case was observed for pharynx, unspecified (ICD-8: 149), whereas 4.4 cases were expected, resulting in a SMR of 0.23 (95% CI: 0.01 – 1.27). Hence, the relationship between the number of cases for NPC and PCns in the cohort followed up is quite different from that for the catchment area of the cancer registry.

Combining observed and expected cases for the whole pharynx from the first study report, no dose–response relationship at all can be detected (Table 2). This statement also holds true when NPC is combined with PCns only (data not shown). Whereas the number of NPC cases rose between first analysis and latest follow-up only by about 50%, the number of cases for the whole group buccal cavity and pharynx (ICD8: 140–149) increased from 21 to 89 in this period. Therefore, it would be very helpful if the authors provided results for all other three-digit ICD-categories of the pharynx to verify the hypothesis described and, hence, to clarify the relationship between exposure to FA and the risk of NPC.

**Table 2:** Mortality from pharyngeal cancer by cumulative exposure to formaldehyde (own calculations, based on data from table 5 [5])

Cumulative exposure (ppm-years)	OBS	EXP	SMR (95% CI)
0 (never)	2	1.1	1.82 (0.22 – 6.57)
(0.0, 0.5]	8	3.6	2.22 (0.96 – 4.38)
(0.5, 5.5]	3	4.1	0.73 (0.15 – 2.14)
(5.5, ∞)	2	2.6	0.77 (0.09 – 2.78)
>0 (ever)	13	10.3	1.26 (0.67 – 2.16)

*OBS* number of observed cases, *EXP* number of expected cases, *SMR* standardized mortality ratio

## References

[1] IARC Working Group on the Evaluation of Carcinogenic Risks to Humans (2012). Chemical agents and related occupations. Volume 100 F. A review of human carcinogens.

[2] Hauptmann M, Lubin JH, Stewart PA, Hayes RB, Blair A (2004). Mortality from solid cancers among workers in formaldehyde industries. Am J Epidemiol.

[3] Beane Freeman LE, Blair A, Lubin JH, Stewart PA, Hayes RB, Hoover RN (2013). Mortality from solid tumors among workers in formaldehyde industries: an update of the NCI cohort. Am J Ind Med.

[4] Marsh GM, Morfeld P, Zimmerman SD, Liu Y, Balmert LC (2016). An updated re-analysis of the mortality risk from nasopharyngeal cancer in the National Cancer Institute formaldehyde worker cohort study. J Occup Med Toxicol.

[5] Blair A, Stewart P, O’Berg M, Gaffey W, Walrath J, Ward J (1986). Mortality among industrial workers exposed to formaldehyde. J Natl Cancer Inst.

